# Treatment options of metastatic and nonmetastatic VIPoma: a review

**DOI:** 10.1007/s00423-022-02620-7

**Published:** 2022-08-05

**Authors:** Azadeh Azizian, Alexander König, Michael Ghadimi

**Affiliations:** 1 Department of General, Visceral, and Paediatric Surgery, University Medical Center Goettingen, Robert-Koch-Straße 40, Goettingen, 37077 Germany; 2 Department of Gastroenterology, Gastrointestinal Oncology, and Endocrinology, University Medical Center Goettingen, Goettingen, Germany

**Keywords:** VIPoma, VIP, Treatment, Metastasis

## Abstract

**Purpose:**

VIPoma belongs to the group of neuroendocrine neoplasms. These tumours are located mostly in the pancreas and produce high levels of vasoactive intestinal peptide (VIP). In most cases, a metastatic state has already been reached at the initial diagnosis, with high levels of VIP leading to a wide spectrum of presenting symptoms. These symptoms include intense diarrhoea and subsequent hypopotassaemia but also cardiac complications, with life-threatening consequences. Treatment options include symptomatic therapy, systemic chemotherapy and targeted therapy, as well as radiation and surgery. Due to the low incidence of VIPoma, there are no prospective studies or evidence-based therapeutic standards to date.

**Methods:**

To evaluate the possible impact of different therapy strategies, we performed literature research using PubMed.

**Results:**

All possible treatment modalities for VIPoma have at least one of two therapy goals: antisecretory effects (symptom control) and antitumoural effects (tumour burden reduction). Symptomatic therapy is the most important in the emergency setting to rehydrate, balance electrolytes and stabilise the patient. Symptomatic therapy is also of great importance perioperatively. Somatostatin analogues play a major role in symptom control, although their efficiency is often limited. Chemotherapy may be effective in reaching stable disease for a certain time period, although its impact on symptom control is limited and often delayed. Among targeted therapy options, the usage of sunitinib appears to be the most effective in terms of symptom control and showing antitumoural effects at the same time. Experience with radiation is still limited; however, local ablative procedures seem to be promising options. Peptide receptor radiotherapy (PRRT) with radiolabelled somatostatin analogues (SSAs, 177Lu-DOTATATE) offers a targeted approach, especially in patients with high somatostatin receptor density. Surgery is the first-line therapy for nonmetastatic VIPoma. Additionally, if the resection of all visible tumour lesions is possible, the surgical approach seems preferable to other strategies in highly symptomatic patients. The role of surgery in very advanced stages where only tumour debulking is possible remains debatable. However, a high rate of immediate symptom control can be achieved by tumour debulking followed by somatostatin therapy, although the impact on survival remains unclear.

**Conclusion:**

Surgery is the only curative option for nonmetastatic VIPoma. Additionally, surgery should be a first-line therapy option for highly symptomatic patients, especially if the resection of all tumour lesions (primary tumour and metastasis) is achievable. In frail patients, other modalities can be used.

## Introduction

Vasoactive intestinal peptide (VIP), first discovered in 1966 [1], is a gastrointestinal peptide hormone encoded by the VIP gene on chromosome 6 [2]. The peptide is mainly produced in the duodenum and in delta-2-pancreatic islet cells but is also found in central and peripheral neurons. VIP mediates a variety of functions in the human body, including gastrointestinal effects causing severe life-threatening diarrhoea, but also vasocardial and neuronal effects. Furthermore, VIP affects the respiratory system, growth and carcinogenesis and the immune system [3].

**Fig. 1:**
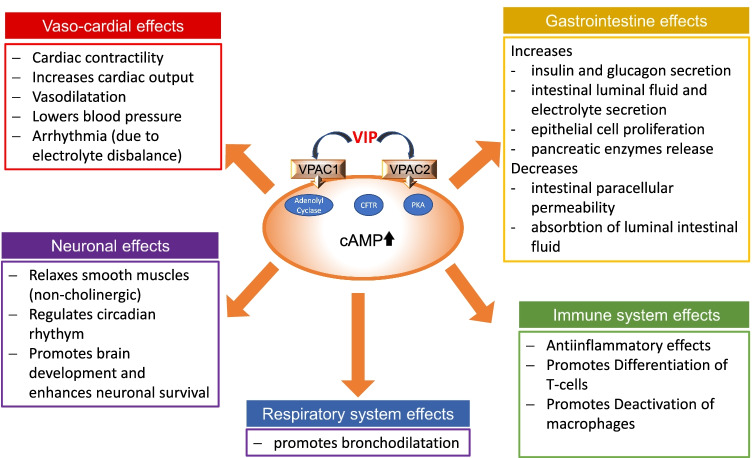
Overview of the effects of VIP in the human body

The half-life of VIP in blood is rather short, as for peptide hormones in general, at only approximately 2 min [4]. VIP stimulates cyclic adenosine monophosphate (cAMP) production in cells, leading to a variety of effects, including increasing intestinal luminal fluid and electrolyte secretion as well as insulin and glucagon secretion and inhibiting gastric acid secretion. Figure 1 shows an overview of the underlying mechanisms and effects of VIP in the human body.

Congruent with its involvement in numerous cellular processes, deregulated high levels of VIP result in a variety of severe symptoms. Elevated serum VIP levels are found almost exclusively in patients with neoplastic neuroendocrine lesions. VIPoma, a neuroendocrine tumour (NET) mostly occurring in the human pancreas, produces high levels of VIP. VIPoma is a rare disease with an incidence rate of approximately 1 case per 10,000,000 person-years. VIPoma is also called *Verner–Morrison syndrome* after its discoverers, Verner and Morrison [5], or *WDHA* as an acronym for its main symptoms (water, diarrhoea, hypokalaemia and achlorhydria). VIPoma was first discovered in 1958 [5], and its pathophysiology was described 15 years later by Bloom et al. [6]. In 1983, Kane and colleagues reproduced VIPoma-like symptoms by intravenous administration of VIP [7]. These symptoms included vasodilation, glycogenolysis, lipolysis and bone resorption as the main effects of high VIP levels in blood. VIPoma also leads to the secretion of water and electrolytes from GI epithelial cells, leading to hypopotassaemia, facial flushing, elevated blood glucose and hypercalcaemia [8].

In adults, most VIPomas originate from the pancreas and arise without known germline genomic alterations. However, in approximately 5% of all cases, VIPomas may also be associated with multiple endocrine neoplasms [9]. In paediatric patients, VIPomas can originate from sympathetic nervous system ganglia [10]. Nonneurogenic, extrapancreatic VIPomas have rarely been described in case reports [11, 12].

Usually, patients with VIPoma present with severe and life-threatening clinical symptoms such as diarrhoea, exsiccosis or hypopotassaemia, resulting from excessive hormone production in an already metastatic state of the disease. These severe complications usually make immediate therapeutic decisions inevitable. Stabilisation, rehydration and balancing electrolytes are of the utmost importance when taking measures in an emergency setting to avoid severe consequences. Antisecretory therapy and antitumoural therapy are the main treatments of VIPoma patients. Treatment options include symptomatic therapy, chemotherapy, radiation, local therapeutic approaches such as chemoembolisation or radiofrequency ablation and surgery.

Due to its rarity and the lack of large cohort studies, there are no consensus management guidelines for treatment. In the nonmetastatic stage, surgical resection is undoubtedly the only curative therapy. However, in most cases, there is diffuse metastasis at the initial diagnosis of VIPoma. Here, different approaches are possible.

In the following, we aim to present the spectrum of therapy options and modalities, particularly the impact of surgical approaches in metastatic stages, for VIPoma patients, offering an algorithm for therapy decisions based on the existing data and our own experience.

## Methods

A PubMed search of the English literature from January 2000 to January 2022 was performed with the terms ‘VIPoma’, ‘Vasoactive Intestinal Peptide Producing Tumor’, ‘WDHA’, ‘Verner-Morrison-Syndrome’ and ‘pancreatic cholera’. All case reports, case series and retrospective cohort analyses describing presentations of the disease, treatment options and VIP-hormone-related data were included. After that, we checked the articles for relevance.

**Fig. 2:**
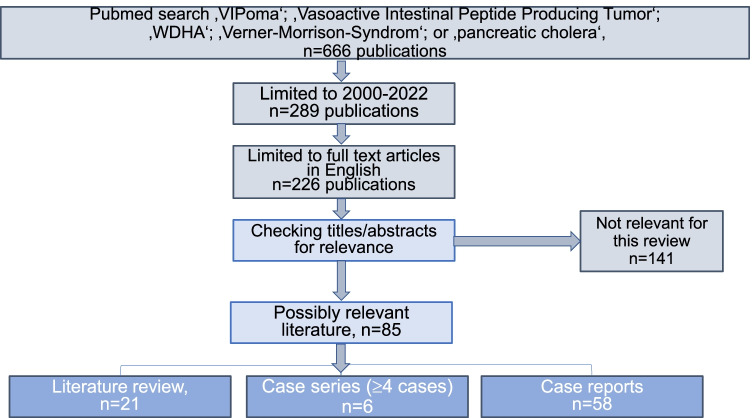
Flowchart describing PubMed research for the present review

Figure 2 shows a flow chart describing the literature search.

## Results

**Fig. 3:**
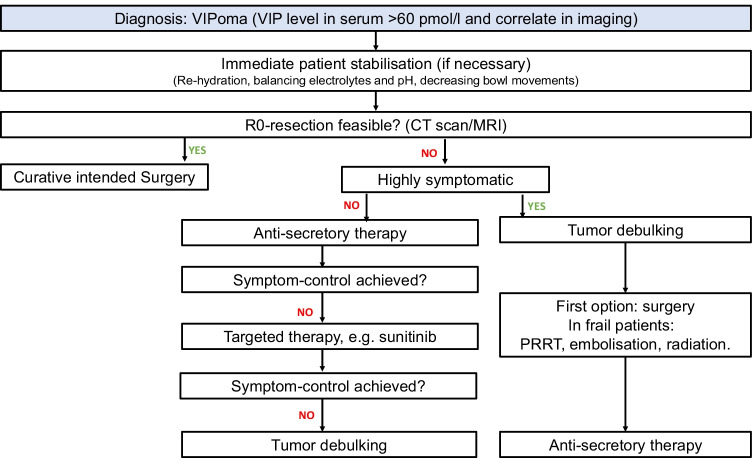
Algorithm for treatment decisions for metastatic and nonmetastatic VIPoma

In approximately 60–80% of all VIPoma cases described to date, the patient presented with metastases at the initial VIPoma diagnosis [13, 14]. The leading symptom is diet-resistant diarrhoea, which can be so severe that the consequences (hypopotassaemia, vasodilatation, anorexia, cramps) might be life-threatening. The therapeutic strategies used can be grouped into ‘antisecretory therapy’ and ‘antitumour therapy’; however, some treatments show both antisecretory effects and antitumoural effects. Although antisecretory treatment is crucial to improve quality of life and in-house mortality, its effects on long-term outcomes have not been explored. Figure 3 presents an algorithm for treatment decisions. Table 1 lists most relevant studies concerning treatment of VIPoma patients with their main results and patient’s outcome.

**Table 1:** List of relevant studies concerning treatment of VIPoma

Author Study design	Year	Country	Patients (*n*)	Main symptoms	Treatment and outcome
Brugel et al Retrospective cohort	2021	France	25	Diarrhoea, vomiting, abdominal pain, dehydration	*In nonmetastatic patients (n* = *4)* Curative-intended surgery (*n* = 3) and neoadjuvant chemotherapy, curative-intended surgery followed by PRRT (*n* = 1): 5-year RFS 60% *In metastatic patients (n* = *21)* Treatment with SSAs alone (*n* = 11), 66.7% significant antisecretory activity Chemotherapy (*n* = 13): median PFS of 9.2 months Transarterial liver embolisation (*n* = 11), symptom control in 50% of all cases, median PFS: 8.6 months Everolimus (*n* = 9), symptom control 20%; median PFS, 11 months Sunitinib (*n* = 7), immediate symptom control 100%; median PFS, 11 months PRRT (*n* = 1), therapy stopped because of poor tolerance Surgery for synchron metastatic VIPoma (*n* = 23) - Resection of primary pancreatic NET (*n* = 11) - Resection of liver metastasis (18 interventions in 12 patients), of those 14 curative-intended (immediate symptom control 100%, median PFS 15.3 months), 4 debulking surgery with palliative intent (immediate symptom control 75%, median PFS 21.1 months
Zandee et al Retrospective cohort	2019	Netherlands	5	Diarrhoea	PRRT: 80% symptom control
Angelousi et al Retrospective cohort	2019	UK	15	Diarrhoea, hypokalaemia	Curative-intended surgery (*n* = 6), immediate symptom control 66%, 2 patients without recurrence (median follow-up 146 months), 4 patients with median PFS of 20 months; SSA (*n* = 13): Chemotherapy (*n* = 5), symptom control in 40%; RFA (*n* = 3), symptom control 100%, median PFS 40 months PRRT (*n* = 6), 80% immediate symptom control; PFS, 26 months Sunitinib (*n* = 5), symptom control 33%; Everolimus (*n* = 1), symptom control 0%
Ghaferi et al Case reports and review	2007	USA	4	Diarrhoea, weight loss hypokalaemia, dehydration	Surgical resection of primary tumour *n* = 4; resection of hepatic metastasis *n* = 2; RFA *n* = 1; one patient died 96 months after surgery
Nikou et al Retrospective cohort	2005	Greece	11	Diarrhoea, hypokalaemia	Surgical resection (*n* = 7), SSA and chemotherapy (*n* = 4), survival data were analysed with associated factors: metastasis and poor differentiation negatively impact prognosis
Peng et al Case reports and review	2004		31	Diarrhoea, hypokalaemia, dehydration	Follow-up in *n* = 11: transarterial liver embolisation (*n* = 2) OS 6 months; curative-intended surgery (*n* = 9) 40–50% survived at least 2 years without recurrence

### Symptomatic therapy

Obviously, adequate symptomatic therapies, including rebalancing electrolytes, sufficient intravenous fluid substitution and diarrhoea treatment, are the first measures to be taken. Due to the possible cardiac effects of electrolyte deregulation [15], close monitoring (e.g. in the ICU) should be considered. In many cases, treatment with somatostatin analogues improves the patient’s situation by decreasing VIP plasma levels and, as a consequence, reducing the high ileal fluid flow [16, 17]. Somatostatin and somatostatin analogues (SSAs), such as octreotide, are a common treatment for all kinds of well-differentiated NENs (as most NEN cells, including VIPoma, express somatostatin receptors on their surface). The application of steroids, clonidine, loperamide or tincture of opium is usually reserved for patients with diarrhoea and those with no response to SSAs. In some patients, the administration of cinacalcet can be used as a treatment for cystic fibrosis transmembrane conductance regulator (CTFR)-mediated secretory diarrhoea. Chemotherapy and targeted therapy are also part of a symptomatic therapy and are described below.

### Chemotherapy

The combination of streptozotocin (STZ) and 5-FU was established as an effective chemotherapeutic option in patients with well-differentiated and moderately differentiated neuroendocrine neoplasia from the pancreas several decades ago [18, 19]. STZ and its combinations have thereby been described as beneficial in oncological tumour growth as well as hormone symptom control. Consistently, these regimens show efficacy in patients suffering from VIPoma and played a central role in treatment strategies in locally advanced or metastatic diseases [20]. Additionally, other combinations of 5-FU with immunomodulatory agents, such as interferon-alpha, have also been described as effective in both tumour growth and hormone symptom control [21]. Nevertheless, the reported median progression-free survival was between 12 [22] and 16.5 months [23], which is not satisfactory, considering the young median age of the patients. The antisecretory effects of chemotherapy are limited and often delayed. For frail patients in whom other antisecretory treatments fail and who are not suitable for surgery, chemotherapy might be a possible course of treatment.

### Targeted therapy

Recent developments in targeted therapeutics have improved medical treatment options in advanced VIPoma. In 2013, Bourcier and Vinik reported improved symptoms and partial response of tumour masses in a 12-year-old boy with metastatic VIPoma after treatment with sunitinib [24], an inhibitor of multiple receptor tyrosine kinases. In 2015, De Mestier et al. described two more cases in which somatostatin-refractory metastatic VIPoma patients were treated with sunitinib [25]. In general, SSA show both antisecretory effects and antitumoural effects in metastatic NET [26]. The discontinuation of the treatment, however, resulted in a sudden recurrence of symptoms, although the exact underlying mechanism remained unclear. Other therapeutics, such as everolimus, cetuximab and rituximab, have been indicated in case reports as possible effective therapeutic options [27]. In a clinical trial for advanced pancreatic neuroendocrine tumours in general, everolimus showed a antisecretory and tumour stabilising effect (in tumours with ≤ 20% proliferation rate) with a median progression-free survival of 11 months (vs. 4.5 months in placebo group) [28]. The most recent case published by Marquez et al. reported a complete response in a 48-year-old female with metastatic VIPoma and insulin cosecretion to a therapy regimen of lanreotide, temozolomide and capecitabine [29]. Others have reported the successful application of this therapy regimen in metastatic NETs in general [22, 23, 30].

### Peptide receptor radionuclide therapy (PRRT)

PRRT remains a newly introduced therapeutic option for NEN patients that targets somatostatin receptor 2 and 5 on the surface of the tumour cells. As shown in the NETTE-1 trial, treatment with ^177^Lu-Dotatate resulted in a significantly longer progression-free survival and a significantly higher response rate than high-dose octreotide LAR in patients with metastatic neuroendocrine tumours of the small intestine [31]. Due to the remarkable objective response rate, PRRT has the potential to reduce tumour load, subsequently hormone-level and finally hormone-dependent clinical symptoms in patients with hormonally active neuroendocrine tumours [32]. Zandee et al. reported an immediate symptom control rate of 80% [33]. Therefore, PRRT remains a potentially valuable therapeutic option in patients suffering from VIPoma.

### Surgery

In general, surgical resection is considered the only curative therapy for nonmetastatic VIPoma [8, 34]. Many case reports have described successful treatment of VIPoma patients via oncological resection of the tumour [35–38], mostly as distal pancreatectomy or pancreatic head resection. Somatostatin or somatostatin analogues are used perioperatively to prevent cardiovascular complications [39]. For metastatic VIPoma, surgical approaches are considered a possible option but not a standard procedure [8]. The ENETS (European Neuroendocrine Tumor Society) guidelines for functional pancreatic neuroendocrine tumours with metastasis state ‘Surgery is generally contraindicated for locally advanced PanNETs when a macroscopic radical resection cannot be achieved’ [40]. However, the recommendations concern functional pancreatic neuroendocrine tumours, which is a very heterogenous group. Regardless, some publications about the resection of metastatic VIPoma reported promising results. For example, Ueda et al. described successful resection of a VIPoma in the pancreatic tail with paraaortic lymph node metastasis in a 72-year-old female [41]. They performed distal pancreatectomy and paraaortic lymphadenectomy, and no recurrence of the disease was detected at the 11-month follow-up. In another case, a 47-year-old male with one hepatic metastasis of a pancreatic tail VIPoma underwent distal pancreatectomy with splenectomy, the resection of the single hepatic lesion and lymph node dissection [42]; the authors also performed radiofrequency ablation for the hepatic lesion postoperatively. During surveillance, the patient was described as being in better health than before surgery. However, residual hepatic lesions were shown on MRI after 6 months, but the patient was still in good health without tumour progression at more than 18 months after the surgery.

In a recent review focusing on clinicopathological data and treatment modalities for pancreatic VIPoma, which included case reports and case series of 65 patients in total, approximately 50% of all patients showed hepatic metastasis; of those, 23.5% received no treatment of the metastasis, 47.1% underwent surgery, and approximately 30% were treated via ablation [34]. Interestingly, among all included patients, the liver was the only site of metastasis. Nonetheless, lung, lymph node, kidney and bone metastases have been reported [10]. Regarding surgical treatment as an option, some authors support surgical resection in metastatic situations with curative intent if the metastasis is completely resectable (e.g. limited to one liver lobe) [8] or as a tumour debulking procedure with palliative intent [43]. A recent case series of 15 VIPoma patients, including 9 with hepatic metastasis at diagnosis, showed that patients who underwent surgery had a longer overall survival than patients who were treated with other therapeutic modalities (44 vs. 33 months) [44].

It is crucial to separate between cases in which all metastases and the primary tumour are resectable and cases in which complete resection of all lesions is not possible (debulking surgery). Brugel et al. showed in a retrospective analysis of liver metastasis resection (*n* = 14) immediate symptom control in 100% of cases, with a median progression-free survival (PFS) of 15.3 months. In *n* = 4 cases, a debulking surgery with palliative intent was performed. Here, an immediate symptom control rate of 75% was achieved with a median PFS of 21.1 months [45].

### Locoregional therapy options

In general, locoregional treatments are used in addition to surgical resection of the tumour. Locoregional treatments seem to be promising options for hepatic lesions smaller than 3 cm. Usage of transarterial chemoembolisation (TACE) for hepatic metastasis of VIPoma is reported in a few case reports [46, 47]. It seems to be a possible alternative to surgery (in frail patients) or as combination with surgery in a two-step approach (first TACE than surgery). In addition to standard locoregional treatments such as TACE and radiofrequency ablation [42, 48, 49], a case report from 2017 suggested the use of percutaneous irreversible electroporation (IRE) as a treatment option [50].

## Conclusion

Due to its rare incidence, there is no standard treatment recommendation for VIPoma, and prospective studies are difficult to carry out. Additionally, VIPomas differ in their grading, Ki67 expression and mitotic rate as well as their plasma VIP level. All of these factors affect patient outcome. Therefore, different approaches in small cohorts are hardly comparable.

Overall, surgery is the gold standard for nonmetastatic VIPoma; for metastatic VIPoma, surgical resection is commonly recommended in several case reports and case series when hepatic metastasis is present. However, some authors do not support surgery for diffuse hepatic metastasis (as a tumour debulking procedure). PRRT is a promising method in patients with a high density of somatostatin receptors. Surgical procedures and additional locoregional treatments (RFA, TACE) as well as antisecretory treatments (SSAs) can be performed in advanced stages, with beneficial effects on the condition of the patient and the option to survive life-threatening symptoms due to high VIP levels. Indeed, surgery could reduce symptoms significantly even when performed as a tumour debulking procedure; thus, the patient may benefit from a chemotherapy-free period. Even curative intent in advanced stages with diffuse hepatic metastasis might also be realistic when the resection or RFA of all lesions is feasible.
